# Collagen Characterization in a Model of Nonalcoholic Steatohepatitis with Fibrosis; A Call for Development of Targeted Therapeutics

**DOI:** 10.3390/molecules26113316

**Published:** 2021-06-01

**Authors:** Anthony J. Pellicano, Kiera Spahn, Ping Zhou, Itzhak D. Goldberg, Prakash Narayan

**Affiliations:** Department of Preclinical Research, Angion Biomedica Corp., 51 Charles Lindbergh Boulevard, Uniondale, NY 11553, USA; apellicano@angion.com (A.J.P.); kspahn5@gmail.com (K.S.); pzhou@angion.com (P.Z.); igoldberg@angion.com (I.D.G.)

**Keywords:** precision therapeutics, NASH, fibrosis, collagen type I, collagen type III, transcriptomic

## Abstract

Left untreated, nonalcoholic fatty liver disease can progress to nonalcoholic steatohepatitis (NASH), fibrosis, and end-stage liver disease. To date, few if any therapies have proven effective against NASH with fibrosis. Quantification and qualification of hepatic scar might enable development of more effective targeted therapies. In a murine model of NASH induced by diet, we characterized fibrillar collagen deposition within the hepatic parenchyma. At harvest, livers from the modified diet cohort exhibited NASH with fibrosis. Transcriptomic analysis of hepatic tissue revealed increased *col1a1, col1a2,* and *col3a1,* each of which correlated directly with hepatic hydroxyproline content. Circular polarized microscopic analysis of Picrosirius red-stained liver sections revealed deposition of collagen type I within the parenchyma. Development of therapeutics designed to mitigate collagen type I accumulation might prove effective in NASH with fibrosis.

## 1. Introduction

Spurred by the diabetes and metabolic syndrome crises, the prevalence of nonalcoholic fatty liver disease (NAFLD) in the United States alone is estimated at 80–100 million individuals [1,2,3]. Left untreated, NAFLD can progress from simple steatosis to nonalcoholic steatohepatitis (NASH), fibrosis, cirrhosis, and hepatocellular carcinoma (HCC) [1,4,5,6]. It is estimated that 8–10 million people in the United States present with NASH and 2–5 million NASH with fibrosis [1]. Within the next two or three decades, NAFLD-related hepatic complications are projected to become the leading cause for liver transplant [1]. To date, there is no approved therapeutic for treatment of NASH with the clinical trials landscape continuing to be beset with failures [7,8,9,10].

Precision medicine involves use of therapies targeting signaling elements driving disease, the premise being that disease drivers might differ amongst patients presenting with the same disease [11]. A therapeutic effective in a given patient might prove ineffective in other patients. A number of reports indicate that deposition of extracellular matrix, i.e., scarring or fibrosis, in the setting of NASH is associated with progression to cirrhosis and/or HCC [4,5,6,12,13,14,15,16]. Identification of transcriptional elements together with identification of the nature of the matrix deposited might yield strategies to more effectively mitigate disease progression. Collagen (COL) types I and III, coded by *col1a1/col1a2* and *col3a1*, respectively, are major fibrillar collagens [17]. COL I promotes HCC by regulating the integrin β1/FAK signaling pathway in NAFLD [18]. In patients with advanced liver fibrosis [19], the COL III formation biomarker PRO-C3 was significantly elevated compared with patients with less advanced disease and independently related to fibrosis stage. These reports point to increased circulating COL I and/or COL III in liver disease, albeit there is no definitive study profiling hepatic COL1 and COL3 in NASH with fibrosis. Even though mRNA expression has already been evaluated in experimental liver fibrosis [20], these markers have not been profiled in a model of NASH.

In a clinically relevant model of diet-induced NASH with fibrosis, the present study tests the hypothesis that liver scarring is associated with increased COL I and COL III genes.

## 2. Results

**Figure 1:**
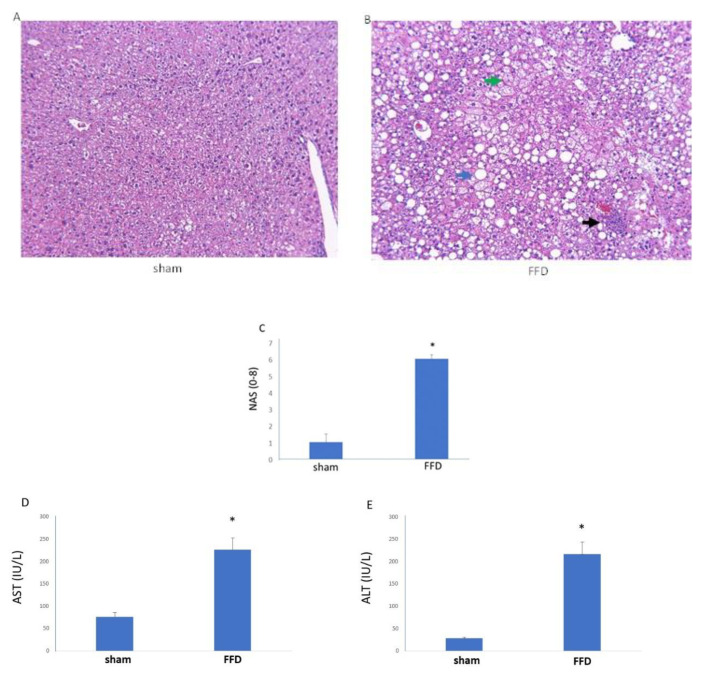
Characteristic Features of Nonalcoholic Steatohepatitis (NASH). Representative images (10× of H&E-stained liver sections from mice randomized to a standard diet (**A**, sham, *n* = 9) or FFD (**B**, *n* =15). The blue arrow shows one of many lipid droplets, the black arrow shows a site of inflammation, and the green arrow shows hepatocyte ballooning. NAS (**C**), AST (**D**), and ALT (**E**) were elevated in the FFD cohort compared with the sham cohort. *, *p* < 0.01.

Compared with livers from the sham cohort, H&E-stained liver sections from the FFD cohort exhibited steatosis, inflammation, and ballooning (Figure 1). Indeed, consistent with a NASH phenotype, NAS, AST, and ALT steatosis were elevated (Figure 1) in the FFD cohort.

**Figure 2:**
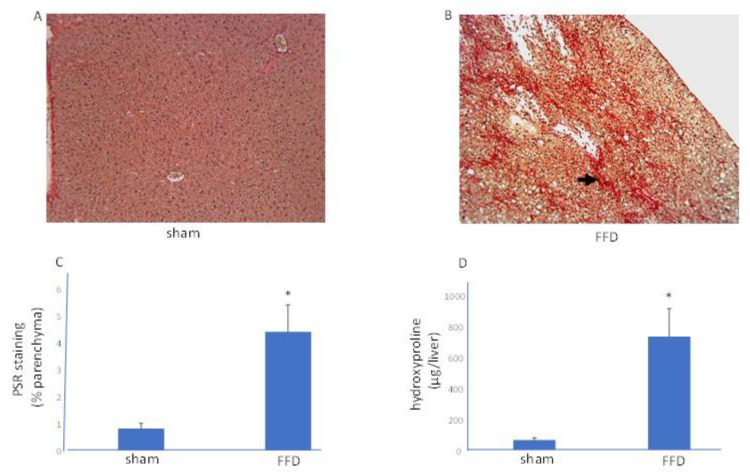
A Model of NASH with Fibrosis. Liver scarring. Representative images (10×) of PSR-stained liver sections from mice randomized to a standard diet (**A**, sham) or FFD (**B**). The black arrow shows bridging fibrosis. (**C**) Quantitation of PSR staining shows elevated scarring in the FFD cohort. (**D**) Compared with the sham cohort (*n* = 9), livers from the FFD cohort (*n* = 15) had increased hydroxyproline content. *, *p* < 0.01.

To determine whether scarring was present in livers from the FFD cohort, PSR staining, which enables visualization and estimation of collagen, was used. A filigree network of PSR staining was evident in these livers with semi-quantification of staining exhibiting a significant increase in parenchymal matrix deposition (Figure 2). Hydroxyproline content, another marker of matrix deposition, was measured in livers from the sham and FFD cohorts. Compared with the sham cohort, biopsies from the FFD cohort exhibited increased hydroxyproline (Figure 2).

**Figure 3:**
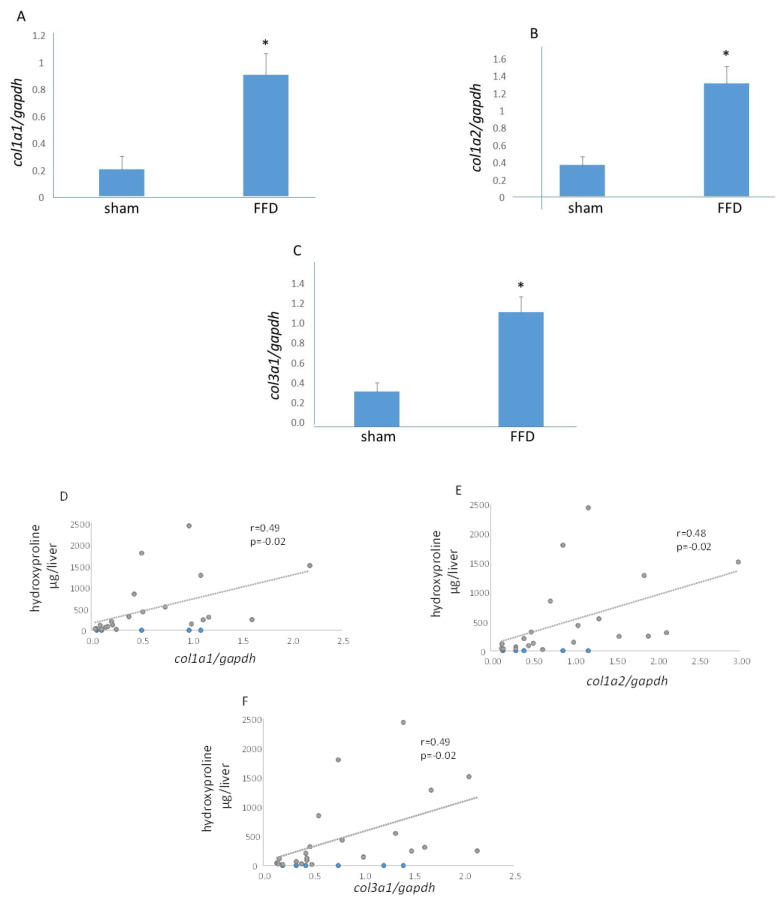
Hepatic *col1 and col3* in NASH with Fibrosis. Increased hepatic transcriptomic expression of *col1a1* (**A**), *col1a2* (**B**), and *col1a3* (**C**) in the FFD cohort; *, p<0.05 vs. sham. Hepatic *col1a1* (**D**), *col1a2* (**E**), and *col1a3* (**F**) expression each exhibited a direct correlation with liver hydroxyproline content.

Liver homogenates were queried for levels of the *col1a1, col1a2,* and *col3a1*. Compared with the sham cohort, livers from the FFD cohort exhibited 4.5-, 2.6-, and 3.2-fold increases in *col1a1, col1a2,* and *col3a1*, respectively (Figure 3). Furthermore, as hypothesized, direct and significant correlations were observed between liver hydroxyproline content and hepatic *col1a1, col1a2,* and *col3a1* expression levels (Figure 3).

**Figure 4:**
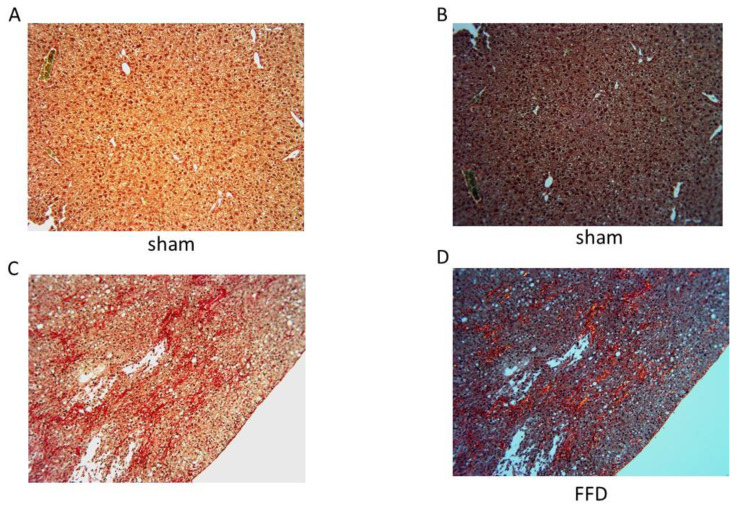
Collagen Deposition in NASH with Fibrosis. Representative images (10×) of a PSR-stained liver section from a mouse randomized to a standard diet under brightfield (**A**, 10×) or circularly polarized light (**B**). Representative images (10×) of a PSR-stained liver section from a mouse randomized to FFD under brightfield (**C**, 10×) or circularly polarized light (**D**) exhibiting yellow-orange birefringence.

Circular polarized PSR microscopy was used to determine the nature of any deposited COL. Bright field images showed characteristic PSR staining in the FFD cohort but not in the sham cohort while under polarized light, in the FFD cohort, PSR staining appeared bright yellow-orange, indicative of COL I deposition (Figure 4) [21,22].

## 3. Discussion

Mice fed an FFD exhibited hallmark features of NASH with fibrosis and increased hepatic expression of *col1a1, col1a2,* and *col3a1.* These gene expression levels exhibited a direct and significant correlation with liver hydroxyproline content. Extracellular matrix deposition in the liver consisted of COL 1 protein.

Left untreated, NAFLD can progress to cirrhosis and decompensated liver failure [1,2,3]. However, the risk associated with NAFLD is not merely limited to the need for transplantation but also includes the risk of developing HCC. In fact, a preponderance of experimental and clinical evidence suggests that increased scar deposition in the liver is associated with transformation of the liver to a cancerous phenotype [4,5,6,12,13,14,15,16]. In murine models of NASH, livers with the highest hydroxyproline content, a component of collagen, exhibited evidence of HCC [4,6]. In fact, in these models liver hydroxyproline content was diagnostic for HCC [6]. Clinically, cirrhotics are at highest risk for HCC. Increasing clinical evidence indicates that NASH with fibrosis can lead to HCC in the absence of cirrhosis, a phenomenon termed noncirrhotic HCC [12,13,14,15,16]. The size of the NAFLD epidemic and the risks associated with the same translate to an urgent need for effective therapeutics against this form of liver disease. Nonetheless, a wealth of regimen effective in preclinical models of NASH have failed to show clinical benefit [7,8,9,10]. The weak translational data may be explained, at least in part, by preclinical evaluation of drug candidates against endpoints including fibrosis. Although laudable, these very endpoints may be a function of different mechanisms based on disease etiology or a subject’s genetics [11]. A drug that effectively mitigates fibrosis secondary to a particular mechanism of action might be ineffective against fibrosis secondary to a different mechanism of action. Better characterization of scarring in NASH might yield targeted therapies that are more effective in mitigating disease or at least mitigating disease in a subset of patients.

Collagen is the principal component of extracellular matrix or a scar [17]. In the present study, we tested the hypothesis that NASH with fibrosis is associated with upregulation of hepatic COL I and COL III, both of which are fibrillar collagens. Consistent with previous reports [4,5,6], livers from mice randomized to a FFD exhibited hallmark features of NASH with fibrosis, i.e., lipid deposition, inflammation, hepatocyte ballooning, increased serum AST and ALT, and matrix deposition. The novel finding in this study was increased hepatic *col1a1, col1a2,* and *col3a1* accompanying NASH with fibrosis. Although the correlation was direct, and significant, the r value was modest, which could be a reflection of the sample size or the genetic machinery being gradually turned off since significant scar had been deposited. Nevertheless, these data are consistent with previous reports [18,19] implicating a role for COL 1 and COL III in NASH. COL I promotes HCC by affecting the integrin β1/FAK signaling pathway in NAFLD. Indeed, HepG2 (a hepatic cell line) cells cultured in matrix prepared from human fatty livers had a higher proliferation rate than those cultured in matrix prepared from normal human livers [18]. Furthermore, HepG2 cells cultured on COL I-coated plates grew more rapidly compared with those on either COL IV- or fibronectin-coated plates [18]. The COL 3 formation biomarker PRO-C3 was significantly higher in patients with advanced fibrosis stage 3–4 than those with fibrosis stage 0–2 [19]. Elevated PRO-C3 levels were also associated with severe lobular inflammation and ballooning. Multivariate logistic regression analysis identified PRO-C3 to be independently related to fibrosis stage. PRO-C3 showed similar performance to identify patients with advanced fibrosis in discovery and validation cohorts. Furthermore, in a longitudinal study cohort with paired biopsies, mean PRO-C3 increased with worsening of fibrosis and decreased with fibrosis improvement [19]. However, the source of the serum PRO-C3 levels remains to be determined. To date there is no definitive study profiling hepatic COL1 and COL3 in NASH with fibrosis. Findings from the present study are novel as there is little doubt regarding the source of these mRNA as the liver, and serum was not queried. Another principal finding from this study was that despite similar upregulation of the COL I and COL III genes, only COL 1 deposition was observed in the liver parenchyma. The PSR stain is one of the best understood histochemical techniques able to selectively highlight collagen networks. Relatively inexpensive, the technique relies on the birefringent properties of collagen molecules. While the PSR stain alone does not selectively bind the collagen network, it becomes more specific than the other common collagen stains when combined with polarized light detection [21,22]. Under circularly polarized PSR microscopy, thin bundles of collagen type III appear yellow-green, whereas large bundles of tightly packed, well-organized, and mature collagen type I molecules appear yellow-orange [21]. Use of circularly polarized PSR microscopy revealed bundles of yellow-orange collagen. Nevertheless, the mechanism underlying this differential deposition remains to be investigated.

This study does have weaknesses in that findings are made from and therefore potentially restricted to this murine model of NASH made at a single time point. Gene expression of other COLs may increase later. Similarly, COL III deposition may be delayed in this model. Nevertheless, given that NASH is a biopsy-driven label [3], characterization of the nature of scar from biopsy can yield valuable information toward management of the patient and development of therapies aimed at promoting COL1 degradation. Another weakness of this study is that the role of other signaling and/or matrix elements including matrix modifying metalloproteinases (MMPs) have not been investigated. Although the precise mechanism-of-action of MMPs in various liver-related disorders remains largely unknown, data from diverse experimental models indicate that these proteinases greatly influence the amount of scar deposited [23]. Furthermore, since collagen accumulation and fibrosis development are only possible if fibronectin is available, the underlying mechanism of potential interest could be a change in fibronectin as was shown by suppressing fibronectin accumulation [24,25,26,27]. Based on the increase in hydroxyproline and collagens in our model, it will be of interest in future studies to evaluate fibronectin too. Nevertheless, these data form the basis for potential use of precisely directed strategies for management of NASH.

## 4. Material and Methods

### 4.1. Animal Model

All in-life studies were conducted in adult male or female C57BL/6 mice (18–20 g, ~6 weeks old) after approval (#2019-014) from our Institutional Animal Care and Use Committee (IACUC). Food and drink were provided ad libitum.

The sham cohort comprised animals (*n* = 9) on a standard rodent diet (5001, LabDiet, St. Louis, MO, USA) for 17 mo. The fast-food diet (FFD) cohort comprised mice (*n* = 15) on a modified rodent diet containing 40 kcal% fat, 20 kcal% fructose, and 2% cholesterol (D09100301, Research Diets, New Brunswick, NJ, USA) for 17 mo [4,5,6]. At sacrifice, animals were anesthetized with ketamine/xylazine (25/5 mg/kg, IP), blood was withdrawn, and livers were harvested.

### 4.2. Histopathology

Histopathological analysis was conducted by an observer blinded to the identity of the groups from the sham (*n* = 9) and FFD cohorts (*n* = 15). Formalin (10%)-fixed liver tissue stained with hematoxylin and eosin (H&E) was evaluated for steatosis, inflammation, and ballooning, and the NAFLD activity score (NAS) was computed [4,5,6]. This scoring system on the 0–8 scale (8 being most diseased) totals the individual component scores for steatosis (0–3), lobular inflammation (0–3), and hepatocyte ballooning (0–3). Picrosirius red (PSR)-stained liver sections were semi-quantified (ImageJ) by a blinded observer and averaged for each liver to estimate extracellular fibrillar collagen. Several fields per liver were evaluated to ensure that the data were representative of that liver.

### 4.3. Liver Function Tests and Hepatic Hydroxyproline

Serum samples were sent to Northwell Health (Lake Success, NY, USA) for determination of aspartate aminotransferase (AST) and alanine aminotransferase (ALT).

Hepatic hydroxyproline content was evaluated [21] in liver sections snap-frozen in liquid N_2_ and stored at −80 °C until analysis. Tissue was weighed and then homogenized in 500 μL of water. Five hundred microliters of HCl (10.0 N) was added to the samples and hydrolyzed at 120 °C for 3 h. Supernatants were transferred to a 96-well plate, and wells were allowed to evaporate dry. Hydroxyproline content was determined by colorimetric (catalog # MAK008, Sigma Aldrich, St. Louis, MO, USA) analysis and expressed as µg hydroxyproline/mg liver.

### 4.4. Hepatic col1a1, col1a2, and col3a1 and COL Phenotyping

RNA isolation was performed using the RNeasy Mini Kit by Qiagen and the manufacturer’s protocol was followed. Liver tissue (~10 mg) was added to 350 μL of RLT buffer and 10 μL of ALI BME. One millimeter diameter zirconium-oxide beads were then added to the tubes and placed into a Next-Advance Bullet-Blender Storm 24 bead homogenizer, where the solution was homogenized. The liquid was then poured into a new tube where it was spun inside of a centrifuge. The centrifuged tubes were transferred into new tubes so that 350 μL of ethanol could be pipetted into the mix. The tube was centrifuged before the RNA was pipetted onto a thermoscientific NanoDrop Lite spectrophotometer, where the quantity and quality (A260/A280) of the RNA was measured. Following the Applied Biosystems High Capacity cDNA Reverse Transcription Kit (Catalog # 4368814, Bohemia, New York, USA) manufacturer protocol, the RNA samples were converted into cDNA. Quantitative polymerase chain reaction (qPCR) was then performed on the cDNA in triplicate with the Applied Biosystems TaqMan Fast Advanced Master Mix following the manufacturer’s protocol. Analysis was performed for *col1a1,* (Thermofisher TaqMan Gene Expression Assay-ID: Hs00164004_m1), *col1a2* (Thermofisher TaqMan Gene Expression Assay-ID: Hs01028956_m1), and *col1a3* (Thermofisher TaqMan Gene Expression Assay-ID: Dr03126620_m1) and data normalized to the housekeeping gene *gapdh* (Thermofisher TaqMan Gene Expression Assay-ID: Mm99999915_g1). The synthesized cDNA was diluted with water and vortexed, ensuring that the resulting solution was mixed entirely. TaqMan probes were added to 520 μL of TaqMan master mix and a multi-channel pipette was used to dispense the mixture into plate wells. Following the dispersion of the probe and master mix, the cDNA and water mixture was added to the master mix wells and was mixed into the original mixture by pipetting. Once the master mix and cDNA were added to each plate well, the plate was covered with tape and centrifuged. The plates were then placed in the Applied Biosystems QuantStudio Real-Time PCR system where qPCR was run.

Circularly polarized PSR microscopy [22,28] was utilized to observe accumulation of COL and identify its subtype based on the birefringence pattern. Under polarized light, COL I fibers appear yellow-orange and COL III fibers appear yellow-green. 

### 4.5. Data Analysis

Data are expressed as average ± standard error of the mean. Between group (sham vs. FFD) differences were calculated using Student’s *t*-test and a *p* value < 0.05 was assumed to be significant. Correlations between liver hydroxyproline content, AFP, and OPN were made and curve-fit using Microsoft Excel. The p value for the Pearson product moment, r, was calculated using an online tool [29]. A *p* < 0.05 was deemed significant.

## Data Availability

Data are available upon request.
